# Comprehensive molecular profiling of pulmonary pleomorphic carcinoma

**DOI:** 10.1038/s41698-021-00201-3

**Published:** 2021-06-22

**Authors:** Masaaki Nagano, Shinji Kohsaka, Takuo Hayashi, Toshihide Ueno, Shinya Kojima, Aya Shinozaki-Ushiku, Shigeki Morita, Masumi Tsuda, Shinya Tanaka, Toshiya Shinohara, Yuko Omori, Fumiko Sugaya, Hiroaki Kato, Yoshiaki Narita, Jun Nakajima, Kenji Suzuki, Kazuya Takamochi, Hiroyuki Mano

**Affiliations:** 1 Division of Cellular Signaling, National Cancer Center Research Institute, Tokyo, Japan; 2 Department of Thoracic Surgery, Graduate School of Medicine, The University of Tokyo, Tokyo, Japan; 3 Department of Human Pathology, Graduate School of Medicine, Juntendo University, Tokyo, Japan; 4 Department of Pathology, Graduate School of Medicine, The University of Tokyo, Tokyo, Japan; 5 Division of Pathology, Mitsui Memorial Hospital, Tokyo, Japan; 6 Department of Cancer Pathology, Faculty of Medicine, GI-CoRE GSS, WPI-ICReDD, Hokkaido University, Sapporo, Japan; 7 Department of Pathology, Teine Keijinkai Hospital, Sapporo, Japan; 8 Department of Respiratory Medicine, Teine Keijinkai Hospital, Sapporo, Japan; 9 Department of Thoracic Surgery, Teine Keijinkai Hospital, Sapporo, Japan; 10 Department of General Thoracic Surgery, Graduate School of Medicine, Juntendo University, Tokyo, Japan

**Keywords:** Lung cancer, Cancer genomics

## Abstract

Information regarding the molecular features of pulmonary pleomorphic carcinoma (PPC) is insufficient. Here, we performed next-generation sequencing to determine the genomic and transcriptomic profiles of PPC. We sequenced the DNAs and RNAs of 78 specimens from 52 patients with PPC. We analyzed 15 PPC cases to identify intratumoral differences in gene alterations, tumor mutation burden (TMB), RNA expression, and PD-L1 expression between epithelial and sarcomatoid components. The genomic alterations of six cases of primary tumors and corresponding metastatic tumors were analyzed. *KRAS* mutations (27%) were the most common driver mutations, followed by *EGFR* (8%), and *MET* (8%) mutations. Epithelial and sarcomatoid components shared activating driver mutations, and there were no significant differences in *CD274* expression or TMB between the two components. However, PD-L1 was highly expressed in the sarcomatoid component of several cases compared with the epithelial component. Primary and metastatic tumors shared oncogenic mutations among genes such as *KRAS* and *TP53*, and additional alterations including *NOTCH4* mutations were specifically identified in the metastatic regions. Our data suggest that therapies targeting activating driver mutations may be effective for patients with PPC and that immune checkpoint inhibitors of PPC may be recommended after careful assessment of PD-L1 expression in each epithelial and sarcomatoid component.

## Introduction

Pulmonary pleomorphic carcinoma (PPC) is a rare subtype of non-small cell lung cancer (NSCLC) that accounts for 0.4–1.6% of malignant lung tumors^1,2^. According to the 4^th^ edition of the *World Health Organization Classification of Lung Tumors*^3^, PPC is defined as a poorly differentiated NSCLC comprising ≥10% spindle or giant cells. These tumors, which predominantly arise in men who heavily smoke, are characterized by a poor response to cytotoxic chemotherapy and a worse outcome than other types of NSCLC^4,5^. Two studies identified EGFR-activating mutations in approximately 20% of PPCs^6,7^, some of which exhibit a partial response to gefitinib^8^. However, insufficient information is available regarding the molecular features of PPC and effective therapeutic targets.

Many PPCs comprise an admixture of sarcomatoid (spindle or giant cell elements or both) and epithelial components (adenocarcinoma, squamous cell carcinoma, or undifferentiated NSCLC). Previous studies indicated that genomic intratumoral heterogeneity in cancers is one of the leading determinants of treatment failure and drug resistance^9,10^. Intratumoral heterogeneity in large-cell neuroendocrine carcinoma (LCNEC) combined with NSCLC is characterized by a relatively high (71%) median concordance rate of genomic mutations between these components^11^. To our knowledge, however, published studies do not comprehensively define the intratumor heterogeneity of PPC.

Immunotherapies targeting the programmed death-1 (PD-1)/PD ligand 1 (PD-L1) axis yielded promising results for patients with NSCLC, and several studies suggest that PD-L1 expression may predict the response to this type of immunotherapy^12,13^. Interestingly, >90% of patients with PPC have PD-L1-positive disease, supporting the conclusion that immunotherapy may serve as a potential option for this patient population^14^. However, this study^14^ demonstrates higher levels of PD-L1 in sarcomatoid vs epithelial components, and the potential effect of this intratumoral difference in PD-L1 expression on treatment efficacy is unknown. Further, a high tumor mutation burden (TMB) serves as a biomarker of the tumor response to PD-1/PD-L1 targeted-immunotherapy^15,16^. However, little is known about the effect of the TMB in PPC.

Here we performed next-generation sequencing to analyze the molecular profiles of PPCs. For certain PPC samples, we extracted genomic DNA and RNA from the sarcomatoid and epithelial components and compared them to detect intratumoral differences in gene mutations, RNA expression, and PD-L1 expression. We further compared the gene alterations in several cases between primary tumors and the corresponding metastatic tumors.

## Results

### Patients’ characteristics

The demographic features of 52 patients with PPC are described in Table 1. Their median age at the time of sample collection was 68 years (range, 36‒84 years), 43 (83%) were male, and 47 (90%) were smokers. The median primary lesion diameter was 4.6 cm (range, 1.5–10.3 cm). Their pathological stages were as follows: stage I, *n* = 15 (29%); stage II, *n* = 17 (33%); stage III, *n* = 15 (29%); and stage IV, *n* = 5 (10%). Forty-nine patients underwent surgical resection, and 44 (90%) underwent lobectomy.

**Table 1:** Demographic features of the 52 patients with pulmonary pleomorphic carcinoma.

Feature	No. of patients (*N* = 52)
Median age, years (range)	68 (36–84)
Sex, *N* (%)	
Male	43 (83)
Female	9 (17)
Smoking status, *N* (%)	
Current	31 (60)
Former	16 (31)
Never	3 (5.8)
Unknown	2 (3.8)
Tumor size (cm), median (range)	4.6 (1.5–10.3)
T-stage, *N* (%)	
1	8 (15)
2	18 (35)
3	17 (33)
4	9 (17)
N-stage, *N* (%)	
0	30 (58)
1	11 (21)
2	9 (17)
3	2 (3.8)
M-stage, *N* (%)	
0	47 (90)
1	5 (9.6)
Pathologic stage, *N* (%)	
I	15 (29)
II	17 (33)
III	15 (29)
IV	5 (9.6)
Surgical resection, *N* (%)	49 (94)
wedge	1 (2.0)
segmentectomy	1 (2.0)
lobectomy	44 (90)
pneumonectomy	3 (6.1)
Recurrence after surgery, *N* (%)	
No persistence or recurrence	33 (63)
Recurrence after surgery	14 (27)
Survival status at last census, *N* (%)	
Alive, no evidence of disease	26 (50)
Alive with disease	1 (1.9)
Died of disease	17 (33)
Died of other cause	8 (15)
Follow-up time in months, median (range)	29 (0.5–122)

### Genomic alterations in PPC

**Fig. 1:**
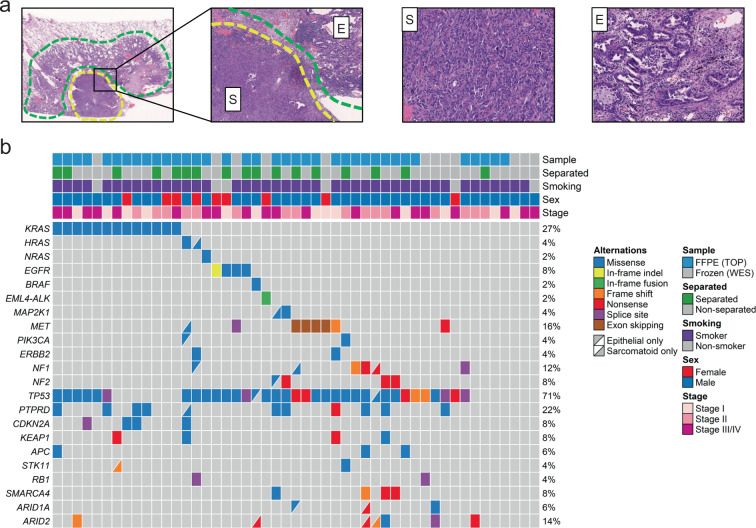
Representative images and genomic alterations of pulmonary pleomorphic carcinomas. **a** This case comprises adenocarcinoma (green area) and sarcomatoid (yellow area) components (hematoxylin and eosin staining). Magnified images of the epithelial (E) and sarcomatoid (S) components are shown. **b** Mutations in *KRAS* were detected in 13 patients (27%). Other activating mutations were detected in *EGFR* (8%), *HRAS* (4%), *NRAS* (2%), *BRAF* (2%), and *MAP2K1* (4%). *MET* exon 14 skipping (4%) and *EML4-ALK* fusion (2%) were detected using RNA sequencing. These driver mutations were mutually exclusive.

The study is summarized in Supplementary Fig. 1. From 4 hospitals, 78 specimens were collected from 52 consecutive patients with PPC in this study. Four FFPE specimens from 3 patients (all were male and current smokers) were excluded from the DNA analysis because of low quality, and 74 specimens from 49 patients were subjected to DNA sequence analyses to detect genomic alterations. Among the 74 specimens, whole-exome sequencing of 12 fresh-frozen tissue samples from 12 patients and target-capture sequencing of 62 FFPE samples from 37 patients were performed. Sixty-six samples were from the primary tumors, and the other eight samples were obtained from the metastatic regions. The genomic DNAs of the sarcomatoid and epithelial components of tumors of 34 samples of 17 patients were individually analyzed (Fig. 1a).

An overview of the mutations in the primary lesions is presented in Fig. 1b. We placed emphasis on determining the genomic alterations associated with lung cancer detected in previous comprehensive genomic studies^17^. *TP53* was the most frequently mutated gene, detected in 35 (71%) patients. *KRAS* mutations (13 patients, 27%) were the most prevalent oncogenic mutations (*G12A*—2 cases, *G12C*—5 cases, *G12D*—1 case, *G12R*—1 case, *G12S*—1 case, and *G12V*—3 cases), followed by *EGFR* (8%), *HRAS* (4%), *MAP2K1* (4%), *PIK3CA* (4%), *NRAS* (2%), and *BRAF* (2%). Other recurrent mutations were identified in *PTPRD* (22%), *ARID2* (14%), and *NF1* (12%). Comprehensive mutation list is shown in Supplementary Data 1.

RNA sequencing of the remaining samples identified an *EML4-ALK* fusion in one patient (2%) and *MET* exon 14 skipping in four (8%) (Fig. 1b). In the sample harboring the *EML4-ALK* fusion, Sanger sequencing of the *EML4-ALK* cDNA revealed that exon 13 of *EML4* was ligated to exon 20 of *ALK* with an insertion of 24 base pairs corresponding to *ALK* intron 19 and six base pairs of unknown origin (Supplementary Fig. 2a). This fusion produces an in-frame transcript with strong oncogenic transforming potential indicated by the focus formation assay (Supplementary Fig. 2b).

We further searched for genomic alterations using the Memorial Sloan Kettering-Cancer Center (MSKCC) cohort data^18^ (http://www.cbioportal.org) (Supplementary Fig. 3). Among 17 cases of PPC, *TP53* and *KRAS* mutations were detected in 10 (59%) and 5 (29%) samples, respectively. These mutation rates were similar to the results of our cohort.

### Comparison of genomic alterations between epithelial and sarcomatoid components

**Fig. 2:**
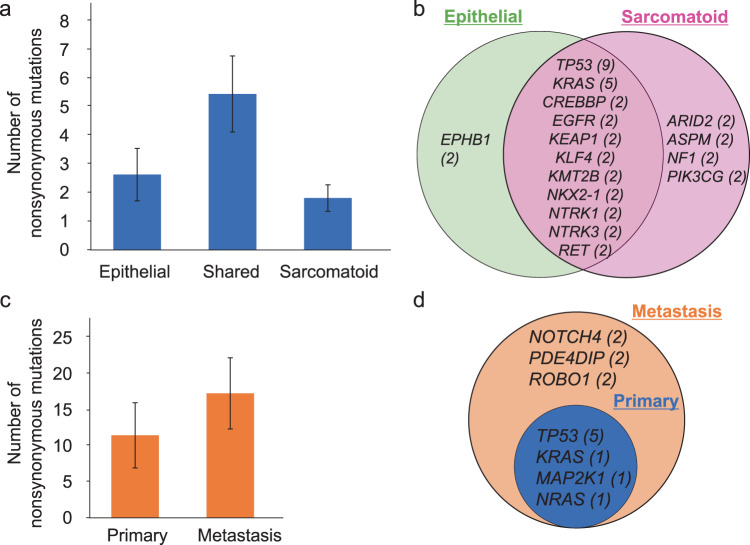
Comparison of genomic alterations according to the intratumoral component and primary or metastatic tissue. **a** Bar chart of the average numbers of shared and unshared nonsynonymous mutations between the epithelial and sarcomatoid components. **b** Individual repeatedly detected mutations in each component. Activating mutations were shared between components, while *ARID2*, *ASPM*, *NF1*, and *PIK3CG* mutations were only detected in sarcomatoid components. The number of patients who harbor the mutations of indicated genes is shown in parentheses. **c** Average numbers of nonsynonymous mutations in primary and metastatic tumors, revealing more in metastatic tumors relative to primary tumors. **d** Individual mutations detected in primary and metastatic tumors. The number of patients who harbor the mutations of indicated genes is shown in parentheses. Error bars, standard error of the mean.

The genomic alterations in epithelial and sarcomatoid components were compared in 17 PPC cases. The mean number of shared nonsynonymous mutations detected in both components was 5.5 (range, 0‒20), whereas the mean numbers of private nonsynonymous mutations (detected in one component) were 2.7 (range, 0‒9) and 1.8 (range: 0‒7) in epithelial and sarcomatoid components, respectively (Fig. 2a). The recurrent nonsynonymous mutations are listed in Fig. 2b. Notably, oncogenic *KRAS* and *EGFR* mutations were shared by both components. Mutations of *ARID2*, *ASPM*, *NF1*, and *PIK3CG* were detected in the sarcomatoid components, while *EPHB1* mutations were observed only in the epithelial components. A phylogenetic tree of each tumor was generated using the LICHeE method (Supplementary Fig. 4)^19^.

### Comparison of genomic alterations between primary and the corresponding metastatic tumors

We determined the differences in genomic alterations between primary and the metastatic tumors of six cases. Notably, a higher number of nonsynonymous mutations were observed in the metastatic tumors (mean, 17.3; range, 3‒28) compared with those in primary tumors (mean, 11.5; range, 1‒33) (Fig. 2c). The oncogenic mutation in *KRAS*, *NRAS*, or *MAP2K1* was identified in one case each, which was detected in the primary and metastatic region. *TP53* mutations were detected in both tumors of five cases. *PDE4DIP*, *ROBO1*, and *NOTCH4* mutations were observed only in metastatic tumors, whereas mutations specific to primary tumors were undetectable (Fig. 2d). Phylogenetic trees of these six cases were generated using the LICHeE method (Supplementary Fig. 5).

### RNA expression profiles in PPC, pathological stages I and II

**Fig. 3:**
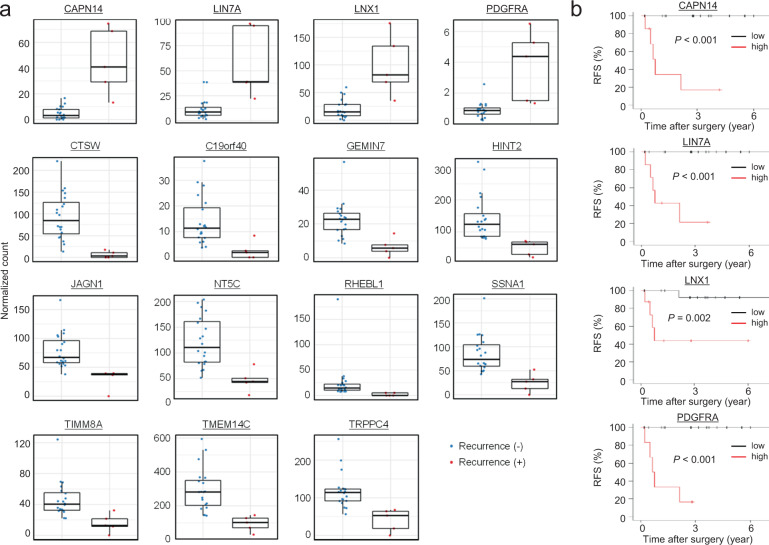
RNA expression in pathological stages I and II pulmonary pleomorphic carcinomas. **a** The expression level of each gene was calculated and normalized using the DESeq2 package. The association between the RNA expression of each gene and recurrence-free survival (RFS) of 25 patients with pathological stage I or II PPC was assessed using univariate Cox proportional hazard regression model, showing the expression levels of the 15 genes strongly correlated with RFS (*q* < 0.05). The midline in each box represents the median, and the lower and upper boundaries indicate the first and third quartiles, respectively. Whiskers represent the 95% confidence intervals of the mean values. **b** Kaplan–Meier analysis of RFS of 25 patients according to the average RNA level of each gene demonstrated that strong expression of four genes was associated with shorter RFS (*p* < 0.05).

Even patients with early-stage PPC face a significant risk of recurrence after undergoing complete surgical resection^1,2^. Therefore, reliable prognostic biomarkers are desirable to identify such patients. For this purpose, association between the RNA expression of each gene and recurrence-free survival (RFS) of patients with pathological stage I or II PPC who underwent complete surgical resection was assessed using a univariate Cox proportional hazards regression model. Among the 32 patients with stage I or II in our cohort, 25 patients were analyzed using FFPE samples and the others were analyzed using fresh frozen samples. Because the difference in starting material can cause difference in the representation of RNA expression, survival analysis was performed using only FFPE samples from 25 patients. As a result, we found that the expression of 15 genes significantly correlated with RFS (*q* < 0.05, Fig. 3a). The patients were then divided into high and low groups according to the average expression level of each gene, and pairwise comparisons of RFS were performed using the log-rank test. High expression of *CAPN14*, *LIN7A*, *LNX1*, or *PDGFRA* significantly correlated with shorter RFS (*p* < 0.05, Fig. 3b). In contrast, high expression of the other 11 genes correlated with longer RFS (Supplementary Fig. 6).

### Comparison of RNA levels between epithelial and sarcomatoid components

**Fig. 4:**
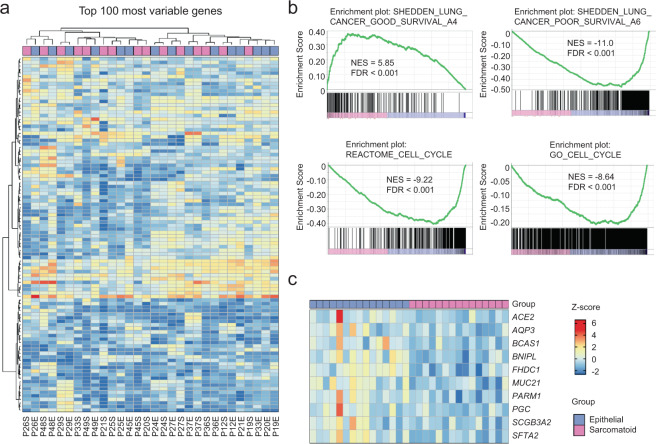
Comparison of RNA expression patterns between epithelial and sarcomatoid components. **a** Heat map of the z-scores of the top 100 genes differentially expressed between the epithelial and sarcomatoid components. **b** Gene Set Enrichment Analysis revealed that the “SHEDDEN_LUNG_CANCER_POOR_SURVIVAL_A6” set was significantly enriched in the sarcomatoid group, whereas the “SHEDDEN_LUNG_CANCER_GOOD_SURVIVAL_A4” set was enriched in the epithelial group. Cell cycle-related gene sets were significantly enriched in the epithelial group. **c** Heat map of the z-scores of 10 genes that were differentially expressed between epithelial and sarcomatoid components. All genes were more strongly expressed in the epithelial group. FDR, false discovery rate; NES, normalized enrichment score.

We next compared the RNA levels of 30 FFPE specimens from each of epithelial and sarcomatoid components of 15 cases. Hierarchical clustering using the most variable 100 genes revealed that the epithelial and sarcomatoid components of respective cases were clustered next to each other, suggesting that the differences among the patients were greater than those between the epithelial and sarcomatoid components (Fig. 4a). Gene Set Enrichment Analysis (GSEA) of the gene sets “SHEDDEN_LUNG_CANCER_POOR_SURVIVAL_A6” and “SHEDDEN_LUNG_CANCER_GOOD_SURVIVAL_A4” revealed that they were enriched in the sarcomatoid or epithelial component, respectively **(**Fig. 4b). GSEA further identified specific and significant enrichment of gene sets related to the cell cycle in the sarcomatoid group. When we searched for biomarkers that distinguished between epithelial and sarcomatoid components using the Wald test, we found that *ACE2*, *AQP3*, *BCAS1*, *BNIPL*, *FHDC1*, *MUC21*, *PARM1*, *PGC*, *SCGB3A2*, and *SFTA*) were significantly expressed only in the epithelial group (*q* < 0.05, Fig. 4c).

### PD-L1 expression and TMB

**Fig. 5:**
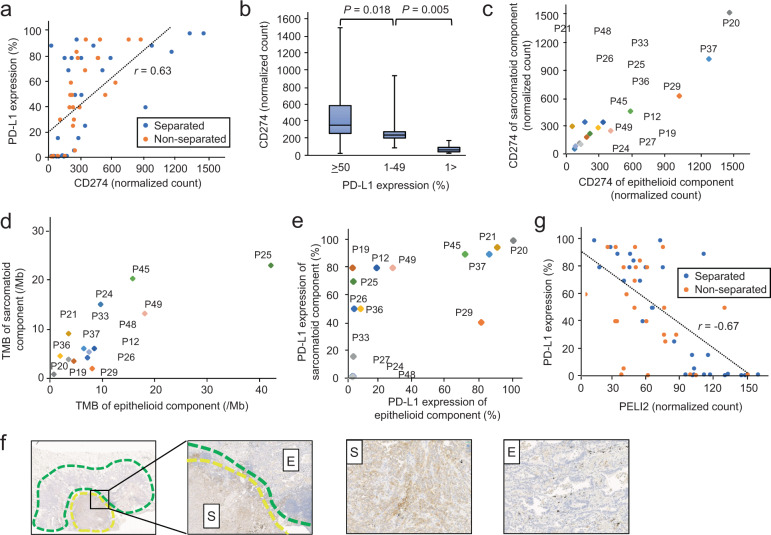
Correlations among *CD274* mRNA expression, tumor mutation burden (TMB), and programmed death ligand 1 (PD-L1) expression. **a** Normalized *CD274* counts significantly correlated with the PD-L1 level (Pearson correlation coefficient *r* = 0.62; *p* < 0.001). Separated and unseparated samples are indicated in blue and orange, respectively. **b** Bar chart of the normalized *CD274* counts according to PD-L1 expression. *CD274* expression was significantly upregulated in the high PD-L1 expression group (≥50%). In each box, the midline represents the median, and the lower and upper boundaries represent the first and third quartiles, respectively. The whiskers indicate the minimum and maximum values. **c** The correlation between the *CD274* normalized counts in the epithelial and sarcomatoid components was evaluated. Strong correlations in the expression of *CD274* were observed between the two components (*r* = 0.95; *p* < 0.001). **d** The correlation between the epithelial and sarcomatoid components with TMB was evaluated. There were strong correlations of the TMB score with each of the two components (*r* = 0.82; *p* < 0.001). **e** PD-L1 levels were compared between the epithelial and sarcomatoid components. Several cases exhibited higher PD-L1 expression in the sarcomatoid component compared with that of the epithelial component. **f** Representative images of immunohistochemical analysis of PD-L1 expression in pulmonary pleomorphic carcinoma. High levels of PD-L1 in the sarcomatoid component (yellow area) were detected, while PD-L1 expression was infrequent in the epithelial component (green area). **g** The Pearson correlation coefficient between the normalized *PELI2* counts and PD-L1 expression was −0.64, the highest absolute value of all tested genes. Separated and unseparated samples are blue and orange, respectively.

When we used IHC to determine the levels of PD-L1 among 56 FFPE specimens compared with those of *CD274* mRNA from the RNA-seq dataset, we found a significant correlation with the latter (Pearson correlation coefficient *r* = 0.63; *p* < 0.001) (Fig. 5a). Using the cut-off thresholds 1% and 50%, according to previous studies^20,21^, we found that the level of *CD274* mRNA was significantly upregulated in the high PD-L1 expression group (≥50%) (*p* < 0.05, Fig. 5b). There was not a significant correlation between the TMB and PD-L1 level in the same specimens (*r* = −0.21; *p* = 0.13) (Supplementary Fig. 7).

The levels of *CD274* mRNA and PD-L1, as well as the TMB, were compared between the epithelial and sarcomatoid components of 15 PPC cases. There was a significant correlation between the level of *CD274* mRNA or the TMB score between the two components (*r* = 0.95; *p* < 0.001 or *r* = 0.82; *p* < 0.001, respectively) (Fig. 5c and d). In contrast, several cases exhibited higher levels of PD-L1 in the sarcomatoid components compared with those in the epithelial components (Fig. 5e and f), indicating that post-transcriptional processes may regulate the levels of PD-L1. We therefore searched for genes whose expression levels correlated with those of PD-L1. The strongest positive correlation was observed for the levels of *CD274* mRNA, supporting the validity of this approach. The strongest negative correlation was observed for the gene encoding Pellino E3 ubiquitin protein ligase family member 2 (*PELI2*, *r* = −0.64) (Fig. 5g). Notably, the correlation coefficient of the association of *PELI2* and PD-L1 was stronger than that between *CD274* mRNA and PD-L1 (absolute *r* = 0.82 vs 0.60) when the analysis separately considered the epithelial and sarcomatoid components.

## Discussion

To the best of our knowledge, this is the first relatively large-scale (*n* = 49) study to conduct a comprehensive evaluation of the molecular profiles of PPCs. The macrodissection analysis of the epithelial and sarcomatoid components of PPCs found that both components harbored similar genomic alterations, including activating driver mutations. Further, the expression level of PD-L1 was more frequently higher in the sarcomatoid components, and the levels of *CD274* mRNA and the TMB were highly concordant between the two components.

Here, we show that 57% (28/49) of the PPC samples harbored activating mutations, which is consistent with the results of other studies^17^. Mutations within members of the *RAS* gene family were particularly frequent (16/49, 33%), suggesting that therapies targeting the RAS pathway may be effective for PPC. For example, ongoing clinical trials targeting KRAS G12 mutations are showing promise for patients with NSCLC^22^. Further investigations of the abilities of RAS-targeted inhibitors to improve the treatment outcomes of patients with PPC are required.

In the present study, our cohort included four patients (8%) with *MET* exon 14 skipping. This frequency was higher than that observed in studies of patients with lung adenocarcinoma (0.9–2.2%)^23,24^, although it was consistent with other reports (3–22%) on PPC^25,26^. Capmatinib and tepotinib are selective inhibitors of the receptor MET, which was recently approved by the United States Food and Drug Administration (FDA) for patients with NSCLC with *MET* exon 14 skipping; therefore, these drugs would be promising for PPC with *MET* exon 14 skipping.

Our present comparison of the epithelial and sarcomatoid components of 17 patients revealed that 11 (65%) carried the same driver mutations in both components. These patients may benefit from therapies specifically targeting *EGFR*, *MET*, and *BRAF* mutations. Moreover, *TP53* and *KEAP1* alterations were detected in both components of numerous cases, suggesting that these alterations occurred early during tumor progression. These results are consistent with studies that analyzed intratumoral heterogeneity of patients with NSCLC^27,28^.

In contrast, private alterations, which likely arose during tumor progression, were identified in 16 patients (94%). Further, private mutations in *ARID2*, *ASPM*, *NF1*, and *PIK3CG* were detected in the sarcomatoid components of multiple cases. *ARID2*, *NF1*, and *PIK3CG* mutations are associated with poor tumor differentiation and the epithelial–mesenchymal transition^29–31^. Thus, private alterations may contribute to the transition to a sarcomatoid phenotype associated with poor prognosis. Moreover, the private alterations in *ASPM* and *ARID2*, whose functions are to regulate the cell cycle^31,32^, may partly explain the enrichment of a gene set related to the cell cycle in the sarcomatoid component.

Primary and metastatic tumors exhibit a high concordance of genomic alterations, including oncogenic mutations of *KRAS* and *TP53*. Two studies of various primary tumors show that matched metastatic regions exhibit a high degree of similarity with respect to genomic alterations^33,34^. Notably, several alterations occur only in the metastatic tumors of PPC, which may be acquired during disease progression. For example, *NOTCH4* mutations promote the metastasis of melanoma cells^35^. The identification of the molecular mechanism of metastasis may lead to the prevention and treatment of metastasis.

The expression of multiple genes may be related to a high risk of recurrence after curative surgery, including patients with early-stage PPC. Here we show that the strong expression of *CAPN14*, *LIN7A*, *LNX1*, and *PDGFRA* was significantly associated with poor prognosis after surgery. *LNX1*, which is strongly expressed in soft tissue sarcoma^36^, contributes to tumor growth by destabilizing p53^37^. Moreover, strong expression of PDGFRA may serve a significant indicator of poor disease-specific survival. PDGFRA regulates mesenchymal cell activity in the tumor microenvironment through mechanisms including vascular reorganization, proliferation, and pericyte recruitment^38,39^. Therefore, FDA-approved drugs targeting PDGFRA, such as regorafenib for colorectal carcinoma and pazopanib for renal carcinoma^40,41^, may inhibit tumor progression of PPC with high *PDGFRA* expression. Few studies evaluated the relationship between cancer progression and *LIN7A* or *CAPN14* expression.

Here we show that PD-L1 was expressed at significantly higher levels in the sarcomatoid components of several PPCs compared with the epithelial components. For example, PD-L1 levels differ among intratumoral the components of PPC^14^. PD-1 expression serves as a predictive biomarker for immune checkpoint inhibitors (ICI)^12,13^. Therefore, treatment with ICIs may eradicate the sarcomatoid component, which is regarded as resistant to chemotherapy. In contrast to the intratumoral heterogeneity in PD-L1 expression, we found that both components had similar TMBs, and thus a TMB score in a portion of a tumor likely represents the TMB of the entire tumor.

The correlation of *CD274* mRNA levels with those of PD-L1 is the subject of at least two studies^42,43^. Notably, in the present study, we found that compared to *CD274* expression*, PELI2* expression was more strongly associated with PD-L1 expression. *PELI2* encodes a member of the E3 ubiquitin ligase family (PELI2) that plays regulatory roles in immune pathways^44^, including promotion of the ubiquitination of IRAK1^45^. IRAK1 promotes the induction of PD-L1 expression by associating with MyD88 and TRAF6 through the IFN-γ and TLR signaling pathways^46,47^. Further research investigating a direct connection of PD-L1 with PELI2 is warranted.

Three limitations of this study must be considered. First, the different tumor components were separately investigated in approximately one-third of samples. Therefore, it was difficult to draw definite conclusions regarding the significance of the comparison of epithelial and sarcomatoid components within PPC. To our knowledge, however, the present study is the first study to separately analyze the PPC components, and thus the results may help to evaluate intratumoral heterogeneity and tumor evolution, which may affect the selection of treatment options. Second, this study lacked sufficient information regarding therapeutic efficacy of molecular targeted drugs and ICIs. Therapeutic efficacy data was not included because half of the tumors did not recur after surgery, and ICI or MET inhibitors have only recently been approved in Japan. Therefore, therapeutic efficacy of these drugs for PPC and its association with biomarkers, such as TMB, PD-L1, or PELI2 expression, should be confirmed in future clinical studies. Third, association between the RNA expression of each gene and RFS was evaluated using univariate analysis in the study because of the sample size; thus, the implications of the findings are limited. Other parameters, which affect RFS, such as lymphovascular invasion, and spread through alveolar spaces, should be included and assessed using multivariate analysis in large future cohort studies.

In conclusion, both epithelial and sarcomatoid components shared activating driver mutations, suggesting that these truncal mutations can be identified by testing either component, and that matched targeted therapy may be effective for PPC patients with druggable mutations. Moreover, there is a significant enrichment for MET exon 14 alterations in PPC, indicating that PPC tumors with negative DNA-based testing for a driver mutation need additional examination by RNA-based testing. Finally, the combination of cytotoxic chemotherapies and ICIs may represent an option for PPC cases without any druggable mutations when they harbor a sarcomatoid component that expresses high levels of PD-L1.

## Methods

### Samples

Tumor specimens were obtained from 52 patients with PPC (surgically resected samples from 49 patients and autopsy tumor samples from three patients) at four Japanese hospitals from 2005 through 2016. However, three cases were excluded because of poor DNA quality. All surgically resected samples were from chemotherapy-naïve patients. Pathological diagnoses were performed by the pathologists A. Ushiku, T. Hayashi, and S. Morita, according to the 4^th^ edition of the *World Health Organization Classification of Lung Tumors*. Pathologic tumor-node-metastasis (TNM) staging was based on the 8^th^ American Joint Committee on Cancer guidelines. The follow-up endpoint was December 31, 2017. The Institutional Review Board (IRB) of the National Cancer Center, Japan approved this study (research project number: 2015-202). Written informed consent was obtained from all participants except those we were unable to contact due to a loss to follow-up or death at registration. For these latter cases, the Institutional Review Board at each participating institution granted permission for the use of existing tissue samples for research purposes. None of the samples used in this study were obtained from patients who had opted out of study participation.

### Genomic DNA extraction and sequencing

Genomic DNA was extracted from 12 fresh-frozen tissue samples of 12 patients using a QIAamp Fast DNA Tissue Kit (Qiagen, Hilden, Germany) and sheared using a Covaris LE220 (Covaris, Woburn, MA, USA). Adjacent normal lung fresh-frozen tissue samples were also extracted from each patient as a source of matched normal DNA. Whole-exome sequencing libraries were prepared from 1 µg of genomic DNA using the Agilent SureSelect Human All Exon Kit v6 (Agilent Technologies, Santa Clara, CA, USA) and sequenced using a HiSeq 2500 (Illumina, San Diego, CA, USA) with the paired-end option. Genomic DNA was extracted from 66 formalin-fixed, paraffin-embedded (FFPE) samples of 40 patients using a GeneRead DNA FFPE Kit (Qiagen). FFPE samples from adjacent normal lung tissue were also extracted from each patient as a source of matched normal DNA. The genomic DNA was separately extracted from 18 PPC samples of cores taken from the sarcomatoid and epithelial components (Fig. 1a). Genomic DNA was fragmented using a KAPA HyperPlus Library Preparation Kit (Kapa Biosystems, Wilmington, MA, USA), and 750 ng of each sample was subjected to target fragment enrichment using a custom target-capturing panel (SureSelectXT Custom Kit, Agilent Technologies). The Todai OncoPanel (TOP) included all exons of 465 cancer-relevant genes^48^. The target capture libraries were sequenced using a HiSeq 2500 with the paired-end option. Raw.fastq files were analyzed using FastQC v0.11.3, and the sequencing reads were mapped to the human reference genome GRCh38 using BWA, Bowtie2 (http://bowtie-bio.sourceforge.net/bowtie2/index.shtml), and NovoAlign (http://www.novocraft.com/products/novoalign/). Samples in which <80% of bases were covered at a depth of 100× were considered low quality and excluded from the analyses. Somatic mutations were called using MuTect (http://www.broadinstitute.org/cancer/cga/mutect) and SomaticIndelDetector (http://www.broadinstitute.org/cancer/cga/node/87). Mutations were excluded if the variant allele frequency (VAF) was <10%, or the number of variant reads was <10. False-positive calls were discarded through visual inspection. Further, the heterogeneity and evolutionary trajectory between primary tumors and paired metastatic tumors were evaluated using the LICHeE method^19^, which was developed in 2015 to construct phylogenetic trees for multiple tumors according to the VAFs of somatic single-nucleotide variants. Using the TOP, the TMB was calculated as the total number of nonsynonymous and synonymous mutations divided by the length of the total target region (3.12 Mb).

### RNA sequencing

Total RNA was isolated from 12 fresh-frozen tissue samples of 12 patients using RNA-Bee (Tel-Test, Gainesville, FL, USA) and purified using an RNeasy Mini Kit (Qiagen). After poly(A)-RNA selection, the library was prepared using 1 µg of each sample and an NEBNext Ultra Directional RNA Library Prep Kit (NEB, Ipswich, MA, USA) and sequenced using a HiSeq 2500 with the paired-end option. Total RNA was isolated from 66 FFPE samples of 40 patients using an RNeasy FFPE Kit (Qiagen) and purified using an RNeasy Mini Kit. RNAs from 18 patients were separately extracted from cores taken from the sarcomatoid and epithelial components. RNA quality was calculated using a 2200 TapeStation (Agilent Technologies). The synthesis of cDNAs and library preparation were performed using a TruSeq RNA Access Library Prep Kit (Illumina) and 300 ng of each sample. The libraries were sequenced using a HiSeq 2500 with the paired-end option. Raw.fastq files were analyzed using FastQC v0.11.3, and read mapping to the reference genome GRCh38 was performed using BWA, Bowtie2 (http://bowtie-bio.sourceforge.net/bowtie2/index.shtml), and NovoAlign (http://www.novocraft.com/products/novoalign/). We excluded samples that did not meet the post-sequencing quality control criteria for a good-quality RNA-seq experiment, namely >50% of housekeeping gene regions (*ACTB*, *B2M*, *GAPDH*, *HPRT1*, *HSP90AB1*, *PPIA*, *RPL13A*, *RPLP0*, *TFRC*, and *UBC*) with >100× coverage. Gene fusions were detected using the deFuse pipeline (https://bitbucket.org/dranew/defuse)^49^. *MET* exon 14 skipping was detected by generating reference sequences of the 3ʹ junction of *MET* exon 13 and the 5ʹ junction of exon 15 and counting split reads that supported 60-mers of the junction.

### RNA expression analysis

The expression level of each gene was calculated and normalized using the DESeq2 package (https://bioconductor.org/packages/release/bioc/html/DESeq2.html). Genes were excluded from our analyses if the maximum number of normalized counts of all samples was <20, or if ≥4 samples had normalized counts = 0. Heat maps of the expression data were created using the pheatmap package (https://cran.r-project.org/web/packages/pheatmap). Ward’s clustering method and correlation distances were used to generate hierarchical clusters of genes and samples from the heatmaps.

### Gene-set enrichment analysis (GSEA)

The log fold-change in the expression level of each gene between the epithelial and sarcomatoid components was calculated using the DESeq2 package. All genes were ranked in descending order according to log fold-change values and analyzed using GSEA version 2.2.0. GSEA PreRanked software was used to calculate the normalized enrichment scores (NES) and false discovery rates (FDRs) of gene sets obtained from the MSigDB database, which are publicly available at http://www.broadinstitute.org/gsea/msigdb^50^. A gene set was considered significantly enriched if its NES had an FDR *q*-value < 0.01.

### Immunohistochemistry (IHC)

FFPE sections (4-µm thick) were subjected to IHC using an antibody directed against the extracellular domain of human PD-L1 (clone 22C3; Dako, Glostrup, Denmark) with 1:50 dilution. The slides were stained using a Dako Autostainer Link 48 platform with an automated staining protocol validated for the PD-L1 IHC 22C3 pharmDx assay. The pathologists A. U. and T. H. used a light microscope to score the percentage of positive tumor cells in each sample.

### Detection of *EML4-ALK*

We obtained the sequences of complete *EML4-ALK* transcripts from clinical specimens by subjecting total RNA extracted from fresh-frozen samples to reverse transcription with SuperScript^TM^ IV VILO (Thermo Fisher Scientific, Waltham, MA, USA) followed by PCR using PrimeSTAR HS DNA polymerase (Takara Bio, Shiga, Japan) for 35 cycles at 98 °C for 10 s, 60 °C for 5 s, and 72 °C for 4 min. The primers used were 5ʹ-GCTTGAATTCACTCTGTCGGTCCGCTGAATGAAG-3ʹ (sense) and 5ʹ-GAATACGCGTTCCCAAGGAAGAGAAGTGAGTGTG-3ʹ (antisense). The PCR products were sequenced using the BigDye Terminator version 3.1 Cycle Sequencing Kit (Applied Biosystems, Foster City, CA, USA) and analyzed using a 3730 ABI capillary electrophoresis system.

### Focus formation assay

The cDNAs encoding GFP, ALK, or EML4-ALK were each inserted into the pcx4 retroviral plasmid. The recombinant plasmids were transduced together with packaging plasmids (Takara Bio) into human embryonic kidney (HEK) 293 T cells to produce recombinant retroviral particles. 3T3 cells grown in 6-well plates were infected with ecotropic recombinant retroviruses in the presence of 4 μg/mL polybrene (Sigma-Aldrich, St. Louis, MO, USA) for 24 h. 3T3 cells expressing various mutant proteins were cultured for 2 weeks in Dulbecco’s modified Eagle’s medium-F12 supplemented with 5% bovine calf serum, 2 mmol/L glutamine, and 1% penicillin/streptomycin (all from Thermo Fisher Scientific). The cells were then stained with Giemsa solution to detect foci. HEK 293 T cells and 3T3 cells were purchased from the American Type Culture Collection (Manassas, VA, USA).

### Statistical analysis

Univariate Cox regression analysis was performed to evaluate the correlation between the expression level of each gene and recurrence-free survival (RFS) of patients with pathological stages I and II PPC. Only genes with *q*-value < 0.05 were considered candidates in the correlation analysis. Recurrence-free survival (RFS) curves were generated using the Kaplan–Meier method and compared using the log-rank test. *P* < 0.05 indicates a significant difference. The log fold-change in the expression level of each gene between the epithelial and sarcomatoid components was evaluated using the Wald test, and differences with *q* < 0.05 indicate a significant difference. The correlation between the normalized count of each gene and the level of PD-L1 in each sample was calculated using Pearson’s correlation, and statistical significance was defined as *q* < 0.05. Furthermore, the correlations of the levels of PD-L1 with the levels of *CD274* mRNA, which encodes PD-L1, and the TMB were evaluated using the Mann–Whitney test, and *p* < 0.05 indicates a significant difference. Statistical analyses were performed using the R platform (version 3.5.1; https://www.r-project.org/) and associated packages.

### Reporting summary

Further information on research design is available in the Nature Research Reporting Summary linked to this article.

## Supplementary information

Supplementary Information

Supplementary Data 1

Reporting Summary

## Data Availability

We have deposited the raw sequencing data under accession number JGAS000297 in the Japanese Genotype-Phenotype Archive (http://trace.ddbj.nig.ac.jp/jga), which is hosted by the DNA Data Bank of Japan.

## Code availability

No unreported custom computer code or algorithm was used to generate the results of this manuscript. The references of all source codes are included within Materials and Methods.
