# Supplementary material for: Comprehensive molecular profiling of pulmonary pleomorphic carcinoma
Source: NPJ Precis Oncol. 2021 Jun 22;5:57. doi: 10.1038/s41698-021-00201-3 (PMC8219709; doi:10.1038/s41698-021-00201-3)
Supplement: Supplementary file 1 — Supplementary Information [file 41698_2021_201_MOESM1_ESM.pdf]

Supplementary Information

Comprehensive molecular profiling of pulmonary pleomorphic carcinoma

Contents:

Supplementary Figure 1-7

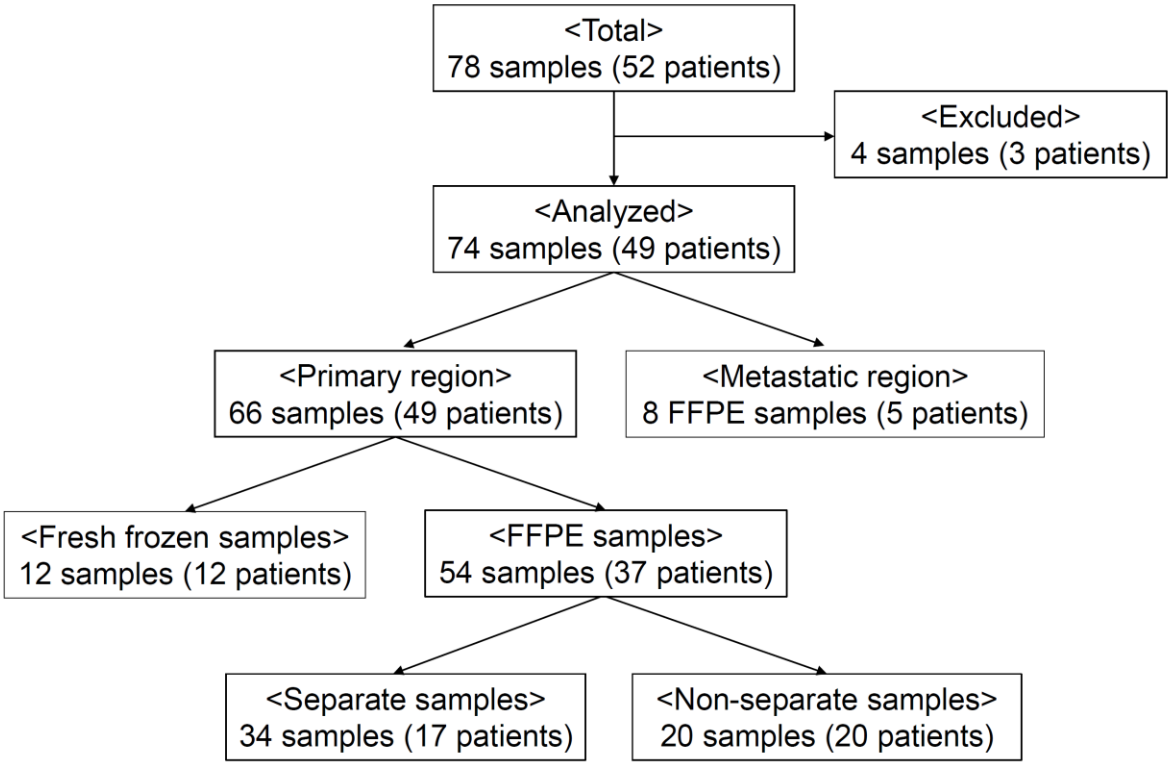

Supplementary Figure 1. Study summary

Four FFPE specimens from three patients were excluded from the DNA analysis because of low quality, and 74 specimens from 49 patients were subjected to DNA sequence analyses to detect genomic alterations. Among the 74 specimens, whole-exome sequencing of 12 fresh-frozen tissue samples from 12 patients and target-capture sequencing of 62 FFPE samples from 37 patients were performed. Sixty-six samples were from the primary tumors, and the other eight samples were obtained from the metastatic regions. The genomic DNAs of the sarcomatoid and epithelial components of tumors of 34 samples of 17 patients were individually analyzed.

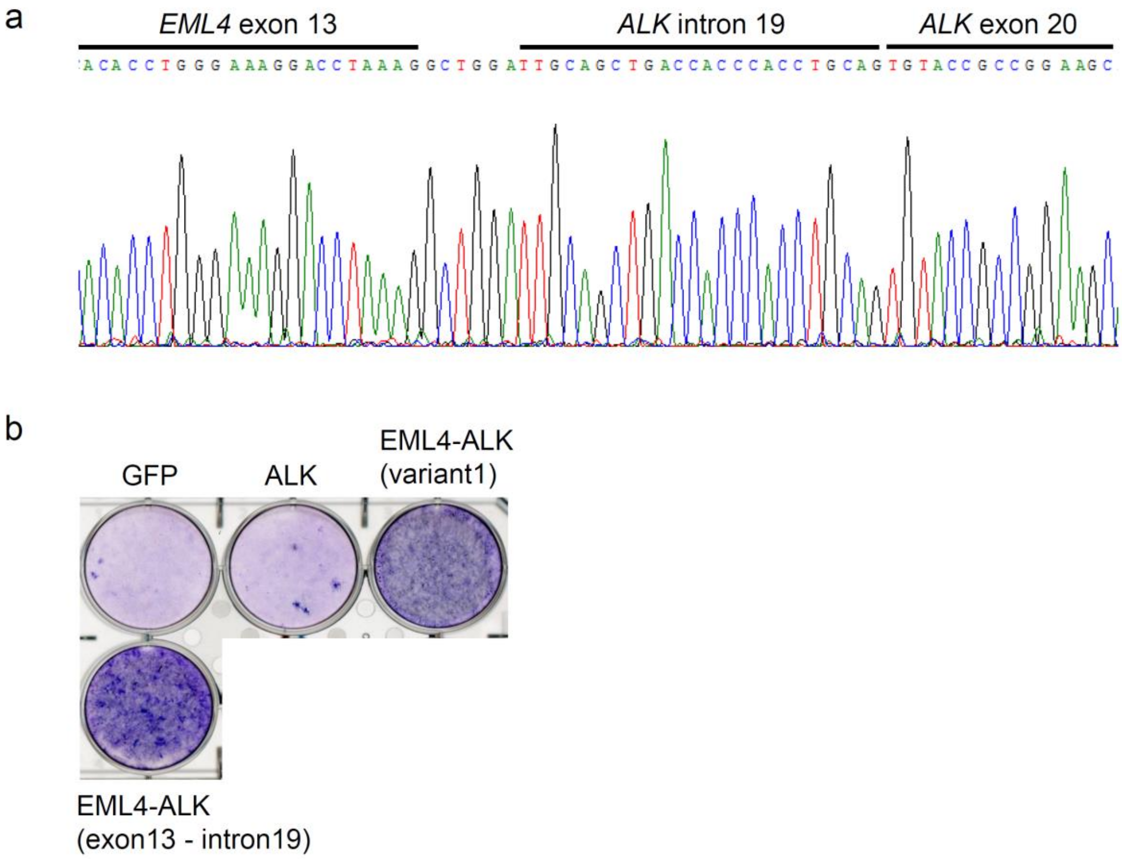

Supplementary Figure 2. Detection of the *EML4-ALK* fusion transcript in pulmonary pleomorphic carcinoma

(a) Sanger sequencing histograms indicate the ligation of exon 13 of *EML4* to intron 19 of *ALK* via an unidentified 6-bp fragment. (b) This EML4-ALK fusion exhibited strong transforming potential in the 3T3 focus formation assay, similar to that of the EML4-ALK variant 1. Three technical replicates for each variant were performed.

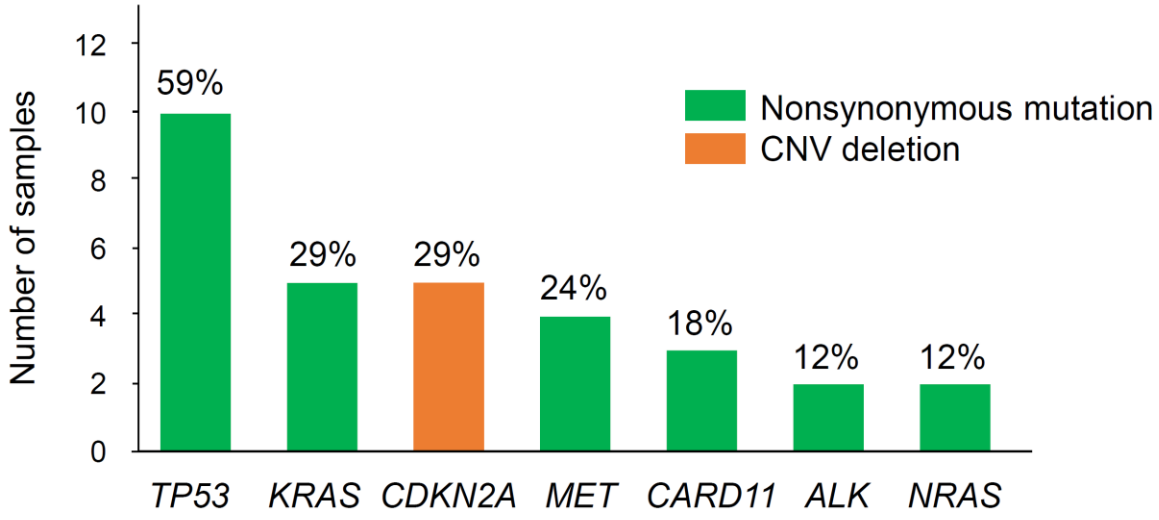

Supplementary Figure 3. Mutation profile of PPC in an MSKCC cohort

Summary of mutations in 17 cases of pulmonary pleomorphic carcinoma (PPC) acquired from cBioPortal. The bar chart illustrates nonsynonymous mutations and copy number variances repeatedly detected in PPC.

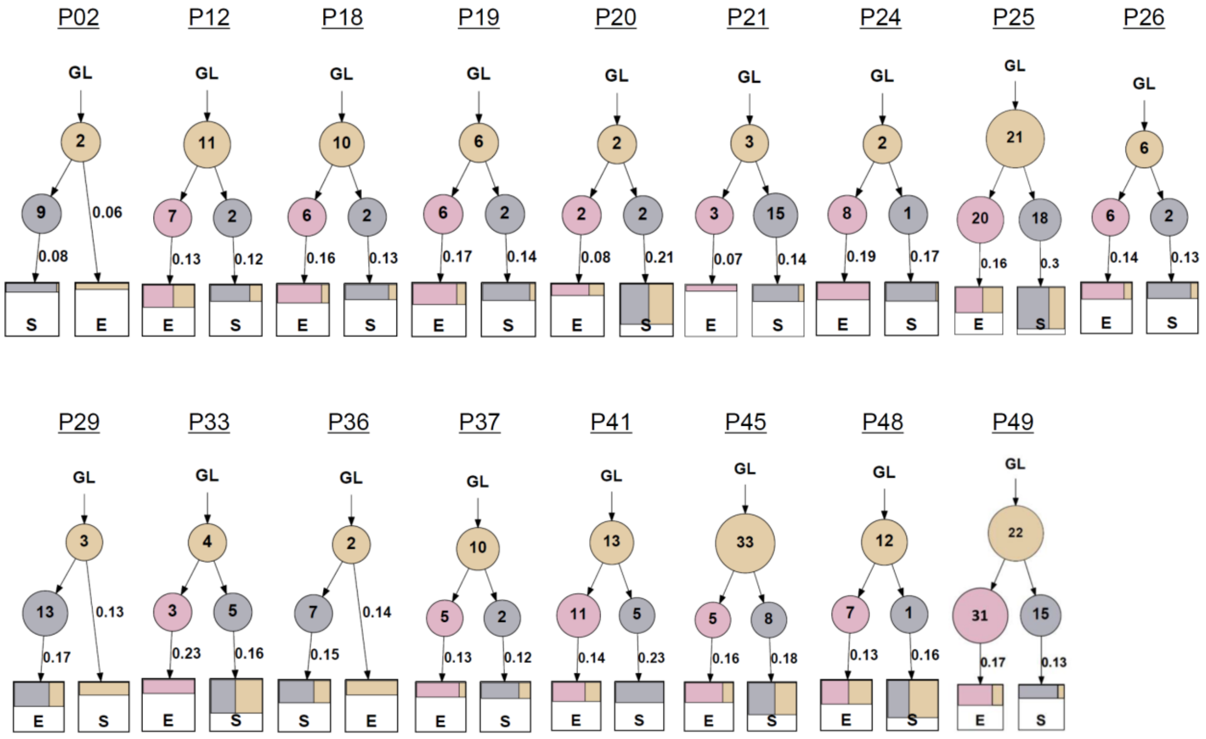

Supplementary Figure 4. Phylogenetic tree of epithelial and sarcomatoid components of PPC

Phylogenetic trees of 17 cases were constructed to compare epithelial and sarcomatoid components using the LICHeE method. The number of shared mutations is in brown nodes while that of private mutations is in pink and gray nodes. E, epithelial component; GL, germ line; S, sarcomatoid component.

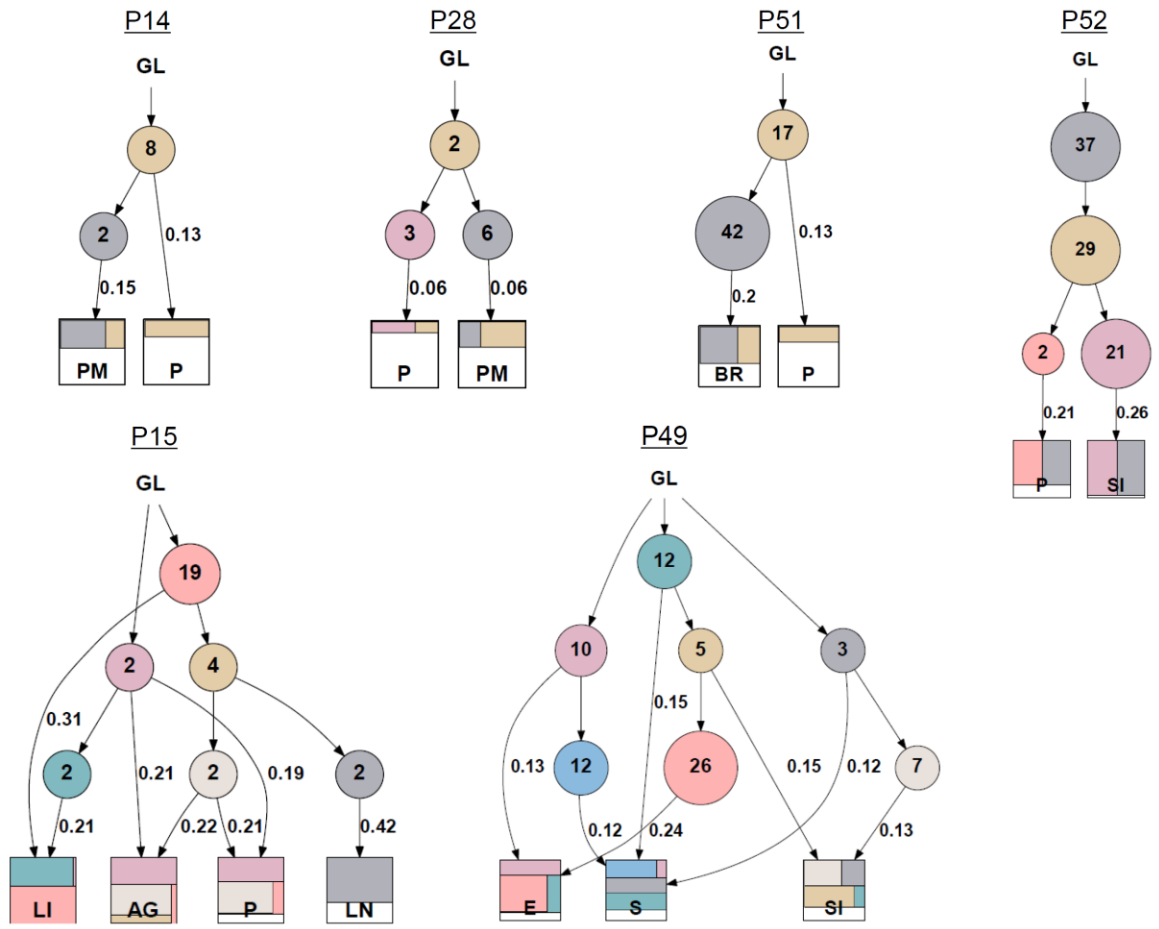

Supplementary Figure 5. Phylogenetic trees of primary and metastatic tumors of PPC

Phylogenetic trees of six patients were constructed to compare primary and metastatic tumors using the LICHeE method. The number of shared or private mutations is shown in the nodes. AG, adrenal grand metastasis; BR, brain metastasis; E, epithelial component (in primary tumor); GL, germ line; LI, liver metastasis; LN, lymph node metastasis; P, primary tumor; PM, pulmonary metastasis; S, sarcomatoid component (in primary tumor); SI, small intestinal metastasis.

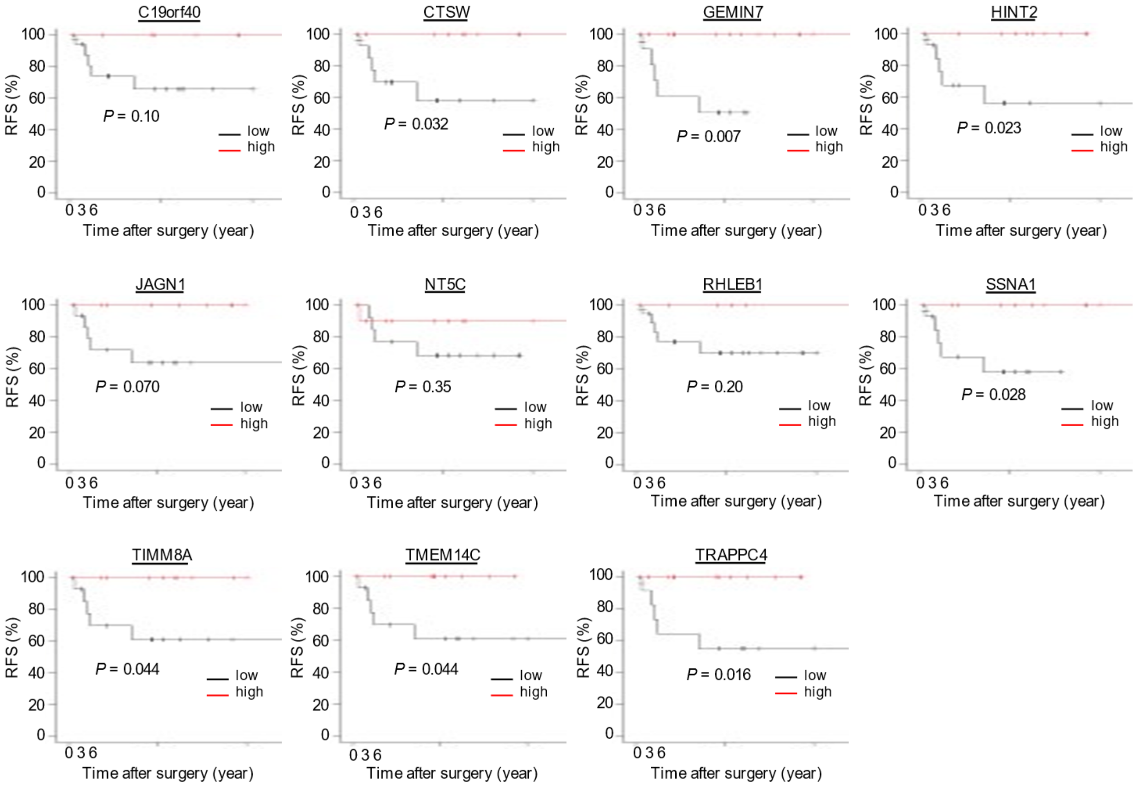

Supplementary Figure 6. Prognostic markers for PPC

Patients with pathological stage I and II were divided into high and low groups according to the average RNA expression level of each gene. Kaplan–Meier curves of recurrence-free survival (RFS) showed that weak expression of 11 genes correlated with shorter RFS.

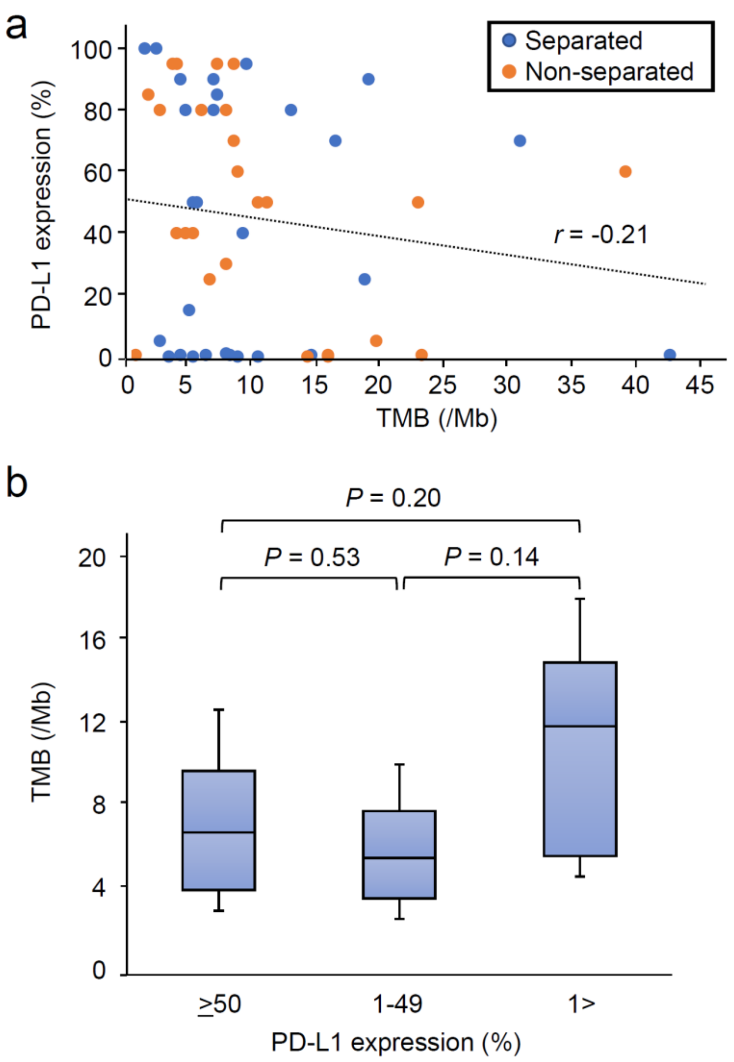

Supplementary Figure 7. Correlation analysis of the tumor mutation burden (TMB) and PD-L1 expression

(a) The TMB did not significantly correlate with PD-L1 expression [Pearson's correlation coefficient (r) = −0.21]. Separated and unseparated samples are blue and orange, respectively. (b) The box plot illustrates the TMB number according to PD-L1 expression and further confirms the lack of correlation between these variables. The box midpoint represents the median, while the lower and upper boundaries represent the first and third quartiles, respectively. The whiskers indicate the minimum and maximum values.
