# Supplementary material for: Comprehensive molecular profiling of pulmonary pleomorphic carcinoma
Source: NPJ Precis Oncol. 2021 Jun 22;5:57. doi: 10.1038/s41698-021-00201-3 (PMC8219709; doi:10.1038/s41698-021-00201-3)
Supplement: Supplementary file 3 — Reporting Summary [file 41698_2021_201_MOESM3_ESM.pdf]

nature research

Corresponding author(s): Shinji Kohsaka
Hiroyuki Mano

Last updated by author(s): 2021/04/24

Reporting Summary

Nature Research wishes to improve the reproducibility of the work that we publish. This form provides structure for consistency and transparency in reporting. For further information on Nature Research policies, see our Editorial Policies and the Editorial Policy Checklist.

Statistics

For all statistical analyses, confirm that the following items are present in the figure legend, table legend, main text, or Methods section.

n/a	Confirmed	
☐	☒	The exact sample size (n) for each experimental group/condition, given as a discrete number and unit of measurement
☐	☒	A statement on whether measurements were taken from distinct samples or whether the same sample was measured repeatedly
☐	☒	The statistical test(s) used AND whether they are one- or two-sided<br>*Only common tests should be described solely by name; describe more complex techniques in the Methods section.*
☒	☐	A description of all covariates tested
☐	☒	A description of any assumptions or corrections, such as tests of normality and adjustment for multiple comparisons
☐	☒	A full description of the statistical parameters including central tendency (e.g. means) or other basic estimates (e.g. regression coefficient) AND variation (e.g. standard deviation) or associated estimates of uncertainty (e.g. confidence intervals)
☐	☒	For null hypothesis testing, the test statistic (e.g. F, t, r) with confidence intervals, effect sizes, degrees of freedom and P value noted<br>*Give P values as exact values whenever suitable.*
☒	☐	For Bayesian analysis, information on the choice of priors and Markov chain Monte Carlo settings
☐	☒	For hierarchical and complex designs, identification of the appropriate level for tests and full reporting of outcomes
☐	☒	Estimates of effect sizes (e.g. Cohen's d, Pearson's r), indicating how they were calculated

Our web collection on statistics for biologists contains articles on many of the points above.

Software and code

Policy information about availability of computer code

Data collection	No unreported custom computer code or algorithm was used to generate the results of this manuscript.
Data analysis	N/A

For manuscripts utilizing custom algorithms or software that are central to the research but not yet described in published literature, software must be made available to editors and reviewers. We strongly encourage code deposition in a community repository (e.g. GitHub). See the Nature Research guidelines for submitting code & software for further information.

Data

Policy information about availability of data

All manuscripts must include a data availability statement. This statement should provide the following information, where applicable:

- Accession codes, unique identifiers, or web links for publicly available datasets
- A list of figures that have associated raw data
- A description of any restrictions on data availability

We have deposited the raw sequencing data in the Japanese Genotype-Phenotype Archive (http://trace.ddbj.nig.ac.jp/jga), which is hosted by the DNA Data Bank of Japan.

Field-specific reporting

Please select the one below that is the best fit for your research. If you are not sure, read the appropriate sections before making your selection.

☒ Life sciences ☐ Behavioural & social sciences ☐ Ecological, evolutionary & environmental sciences

For a reference copy of the document with all sections, see nature.com/documents/nr-reporting-summary-flat.pdf

Life sciences study design

All studies must disclose on these points even when the disclosure is negative.

Sample size	Sample size was described in the manuscript.
Data exclusions	Data exclusions were properly described in the manuscript.
Replication	For next-generation sequencing, no replicate was performed.<br>For focus formation assay, three technical replicates for each variant were performed.
Randomization	Randomization was not performed for this study.
Blinding	Blinding in this study was not performed. Data analysis were performed by the computational pipeline.

Reporting for specific materials, systems and methods

We require information from authors about some types of materials, experimental systems and methods used in many studies. Here, indicate whether each material, system or method listed is relevant to your study. If you are not sure if a list item applies to your research, read the appropriate section before selecting a response.

Materials & experimental systems

n/a	Involved in the study	
☐	☒	Antibodies
☐	☒	Eukaryotic cell lines
☒	☐	Palaeontology and archaeology
☒	☐	Animals and other organisms
☐	☒	Human research participants
☒	☐	Clinical data
☒	☐	Dual use research of concern

Methods

n/a	Involved in the study	
☒	☐	ChIP-seq
☒	☐	Flow cytometry
☒	☐	MRI-based neuroimaging

Antibodies

Antibodies used	A primary antibody against the extracellular domain of human PD-L1 (clone 22C3) was obtained from Dako (Glostrup, Denmark).
Validation	The antibody used in immunohistochemistry was obtained from commercial sources. Please see manufacturer's link for validation of antibody.

Eukaryotic cell lines

Policy information about cell lines

Cell line source(s)	Human embryonic kidney (HEK) 293T cells and 3T3 mouse fibroblasts were purchased from the American Type Culture Collection (Manassas, VA, USA)
Authentication	Cells were not authenticated by the authors.
Mycoplasma contamination	Cells were not tested for mycoplasma contamination.
Commonly misidentified lines (See ICLAC register)	No commonly misidentified cell lines were used.

Human research participants

Policy information about studies involving human research participants

Population characteristics	Tumor specimens were obtained from 52 consecutive patients with pulmonary pleomorphic carcinoma (surgically resected samples from 49 patients and autopsy tumor samples from three patients) at four Japanese hospitals from 2005 through 2016. Three cases were excluded because of poor DNA quality. All surgically resected samples were from chemotherapy-naïve patients.
Recruitment	Chemotherapy-naïve pulmonary pleomorphic carcinoma patients who underwent surgical resection were recruited.
Ethics oversight	The Institutional Review Board of the National Cancer Center, Japan approved this study.

Note that full information on the approval of the study protocol must also be provided in the manuscript.
